# Clinical features and prognosis of pediatric idiopathic sudden sensorineural hearing loss: A bi-center retrospective study

**DOI:** 10.3389/fneur.2023.1121656

**Published:** 2023-03-15

**Authors:** Yingqiang Li, Xiaowei Zhou, Zhiyong Dou, Dongzhou Deng, Dan Bing

**Affiliations:** ^1^Department of Otolaryngology-Head and Neck Surgery, Tongji Hospital, Tongji Medical College, Huazhong University of Science and Technology, Wuhan, China; ^2^Otological Department, The First People's Hospital of Foshan, Foshan, China; ^3^School of Electronic Information and Communications, Huazhong University of Science and Technology, Wuhan, China

**Keywords:** pediatric, audiogram, tinnitus, sudden sensorineural hearing loss (SSNHL), complete blood count (CBC)

## Abstract

**Objective:**

Limited research has focused on the clinical features of sudden sensorineural hearing loss (SSNHL) in pediatric patients. This study is aimed to investigate the relationship between clinical features and the baseline hearing severity and outcomes of SSNHL in the pediatric population.

**Method:**

We conducted a bi-center retrospective observational study in 145 SSNHL patients aged no more than 18 years who were recruited between November 2013 and October 2022. Data extracted from medical records, audiograms, complete blood count (CBC) and coagulation tests have been assessed for the relationship with the severity (the thresholds of the initial hearing) and outcomes (recovery rate, hearing gain and the thresholds of the final hearing).

**Results:**

A lower lymphocyte count (*P* = 0.004) and a higher platelet-to-lymphocyte ratio (PLR) (*P* = 0.041) were found in the patient group with profound initial hearing than in the less severe group. Vertigo (β = 13.932, 95%CI: 4.082–23.782, *P* = 0.007) and lymphocyte count (β = −6.686, 95%CI: −10.919 to −2.454, *P* = 0.003) showed significant associations with the threshold of the initial hearing. In the multivariate logistic model, the probability of recovery was higher for patients with ascending and flat audiograms compared to those with descending audiograms (ascending: OR 8.168, 95% CI 1.450–70.143, *P* = 0.029; flat: OR 3.966, 95% CI 1.341–12.651, *P* = 0.015). Patients with tinnitus had a 3.2-fold increase in the probability of recovery (OR 3.222, 95% CI 1.241–8.907, *P* = 0.019), while the baseline hearing threshold (OR 0.968, 95% CI 0.936–0.998, *P* = 0.047) and duration to the onset of therapy (OR 0.942, 95% CI 0.890–0.977, *P* = 0.010) were negatively associated with the odds of recovery.

**Conclusions:**

The present study showed that accompanying tinnitus, the severity of initial hearing loss, the time elapse and the audiogram configuration might be related to the prognosis of pediatric SSNHL. Meanwhile, the presence of vertigo, lower lymphocytes and higher PLR were associated with worse severity.

## 1. Introduction

Sudden sensorineural hearing loss (SSNHL) is an urgent otologic condition that should be managed promptly. The frequency of SSNHL in adults is expected to range from 5 to 27 cases per 100,000 per year (1), primarily affecting those aged 40–50 years (2–4). Nevertheless, the incidence of SSNHL is 10- to 20-fold lower in children and adolescents than in adults (5), with only 3.5–10% of patients aged < 18 years (6).

To date, there have been a limited number of studies focusing on pediatric patients with SSNHL. It is probably due to the low prevalence. It is common knowledge that children are not “small” adults. Thus, accurately describing the symptoms and relevant medical history is not easy for them. Hearing loss, especially unilateral hearing loss, is sometimes a less perceptible symptom. Thus, it can be difficult to clarify whether a child's hearing loss occurs “suddenly” or in the long run. Another unanswered question is whether SSNHL in children is the same as that observed in adults. Previous study found differences with respect to the recovery rate and audiogram configuration between adult and pediatric patients with SSNHL (6, 7). However, the genesis and progression of SSNHL remain unknown (8). Laboratory tests to explore the etiology, including viral infection (5, 9) and inflammatory biomarkers [neutrophil and lymphocyte count, mean platelet volume (MPV), neutrophil-to-lymphocyte ratio (NLR), and platelet-to-lymphocyte ratio (PLR)] (10–12), were investigated in children, with results that were not entirely similar to those of adults (13–15). Interestingly, we paid more attention to the mental effect of sudden hearing loss on children and their parents. Therefore, data-driven and easy-to-interpret prognostic prediction models are needed to communicate effectively with parents who may be highly anxious to learn about their child's prognosis.

Among the existing studies on pediatric SSNHL, there were some inadequacies. First, the small sample size reduced the power of the statistical analysis. Second, most of the previous studies were conducted in a single center, which made them more susceptible to bias; and third, the statistical methods used were commonly limited to univariate analysis, which was not able to exclude confounding factors to identify adequately independent correlations.

In the present study, we analyzed the clinical parameters associated with the severity and prognosis of SSNHL in children and developed interpretable prognostic prediction models to improve counseling.

## 2. Materials and methods

### 2.1. Study design

**Figure 1:**
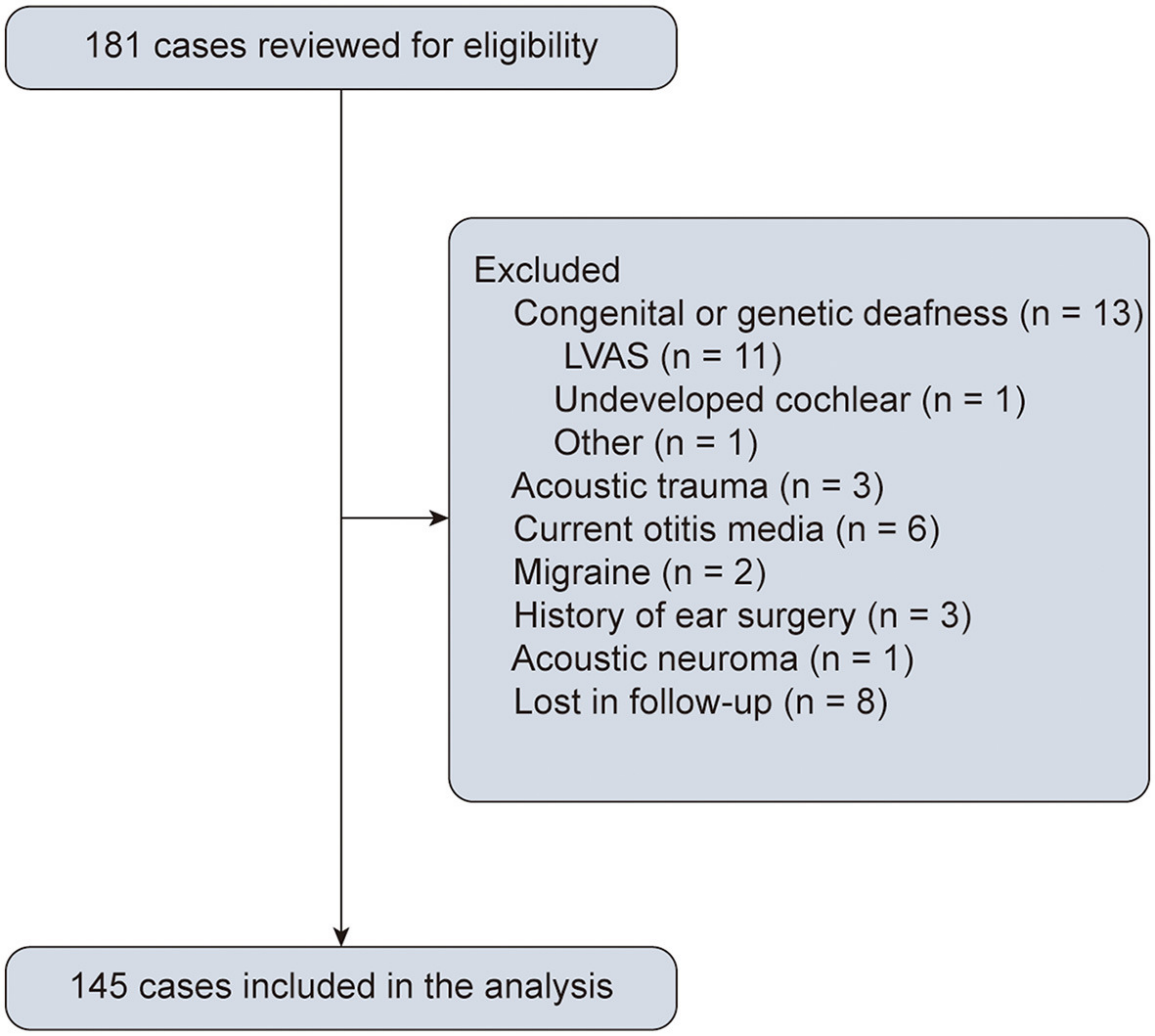
A flowchart depicting the process of study selection through the application of predetermined inclusion and exclusion criteria.

We conducted a retrospective analysis of pediatric patients with SSNHL aged ≤ 18 years who consecutively visited two Chinese tertiary centers (Tongji Hospital, Hubei and The First People's Hospital of Foshan, Guangdong) between November 2013 and October 2022. All the patients underwent a comprehensive clinical assessment, including a neurotological physical examination, audiometry, and imaging. The follow-up visit was conducted in person at the hospital, and 145 patients (85 from Tongji Hospital and 60 from the First People's Hospital of Foshan) were included in the study (Figure 1). We utilized the hospital information system (HIS) and conducted manual screening to collect and review the medical data. The inclusion criteria were as follows: age ≤ 18 years; diagnosed with SSNHL by an otologic specialist based on the criteria outlined in the Chinese guideline (16), i.e. unilateral or bilateral sensorineural hearing loss of >20-decibel hearing levels (dB HL) involving at least two continuous test frequencies developing within 72 h. Participants were excluded if they met any of the following exclusion criteria: (1) had congenital or genetic deafness; (2) had acoustic trauma; (3) had current otitis media; (4) had Meniere's disease; (5) had migraine; (6) had a history of ear surgery; (7) had a history of ototoxic medications; (8) had structural or retrocochlear pathology based on computed tomographic scanning or magnetic resonance (MR) imaging; and (9) were lost to follow-up. The clinical information collected included demographic data, medical records (e.g., the affected ear, accompanying symptoms, time from symptom onset to treatment, and treatment), and laboratory tests (e.g., complete blood count and coagulation tests).

### 2.2. Assessment

The participants enrolled were assessed using pure-tone audiometry. Pure-tone air and bone conduction thresholds at 0.25, 0.5, 1, 2, 4, and 8 kHz were examined based on the standard audiometric methodology. The average baseline hearing threshold was calculated at the affected frequencies of air conduction (frequencies with hearing loss >20 dB HL). The severity of initial hearing loss was classified as follows: mild hearing loss: 20–40 dB HL; moderate hearing loss: 41–60 dB HL; severe hearing loss: 61–80 dB HL; and profound hearing loss: >80 dB HL. We categorized the configurations of the audiogram into four types as described in our earlier study (17): ascending, descending, flat, and cophosis. The results of hearing recovery were estimated using Siegel's criterion (18). Four grades of hearing recovery were referred to as complete recovery (CR, the final hearing level is better than 25 dB HL), partial recovery (PR, the mean threshold for the final hearing levels is between 25 and 45 dB HL and more than 15 dB HL of hearing gain), slight recovery (SR, the final hearing level is worse than 45 dB HL and more than 15 dB HL of hearing gain), and no improvement (NI, < 15 dB HL of hearing gain). Hearing outcomes were also assessed based on the final hearing threshold (calculating the mean of thresholds at the affected frequencies) and hearing gain (the difference between the final and initial hearing thresholds). Before therapy, patients received complete blood counts and underwent coagulation tests. Values of the absolute neutrophil counts, absolute lymphocyte counts, absolute monocyte counts, absolute platelet counts, and mean platelet volume (MPV) were obtained. The absolute neutrophil count divided by the absolute lymphocyte count was used to compute the neutrophil-to-lymphocyte ratio (NLR). By dividing the absolute platelet counts by the absolute lymphocyte count, the platelet-to-lymphocyte ratio (PLR) was determined. The coagulation function parameters included the values of activated partial thromboplastin clotting time (APTT), thrombin time (TT), prothrombin time test (PT), and concentration of fibrinogen. All 145 patients underwent 14 days of treatment containing systemic corticosteroids (prednisolone 1 mg/kg/day, tapered progressively every 4 days) and vasoactive medications.

### 2.3. Statistical analyses

We analyzed the initial severity of hearing loss and hearing outcomes based on Siegel's criterion. The patients with an average baseline hearing threshold graded as profound (>80 dB HL) were allocated to the profound group, whereas those with less severe baseline hearing were assigned to the not profound group. According to Siegel's criterion, patients whose hearing outcomes were classified as CR or PR were allocated to the recovery group, and the remaining patients were assigned to the non-recovery group. The Shapiro–Wilk test was used to determine if a continuous variable was normally distributed. Continuous data with a normal distribution were given as the mean and standard deviation, whereas non-normal data were presented as the median and interquartile range. The frequency and proportion were used to characterize nominal variables. Subgrouping comparisons were performed using appropriate statistical tests, such as the Mann–Whitney *U*-test, Fischer's exact test, and the Student's *t*-test, between the two subgroups. The Kruskal–Wallis test was applied to the ordinal variables for the group comparison. In addition, *post-hoc* pair-wise comparisons were used where there were more than two subgroups. The “glm” package was used to fit multivariate models to explore the factors that affected either the initial hearing or the hearing outcomes. The multivariate linear regression included the baseline hearing threshold as the response variable. Based on the variables having significance in the comparison between groups, the presence of vertigo, the lymphocyte count, and the monocyte count should be incorporated into the model. However, the analysis (Pearson correlation coefficient) revealed a significant linear correlation between the lymphocyte count, the monocyte count, and PLR. Furthermore, when these variables were included in the model, high variance inflation factor (VIF) values were observed. Therefore, we only chose the lymphocyte count to avoid potential collinearity. To analyze factors affecting hearing outcomes, a binomial categorical variable (recovery vs. non-recovery) and a continuous variable (final hearing threshold) were utilized as dependent variables. Significant variables from the univariate analysis for hearing recovery were included in the multivariate linear and logistic regression models, adjusted for age, sex, and time to the onset of therapy. To include nominal variables in the linear regression, a dummy variable was developed with a value of 1 if the case matched the description and 0 otherwise. Regression coefficients, standard regression coefficients, 95% confidence intervals, and odds ratios were calculated using regression models. All analyses were conducted using RStudio (Version: 2022.07.2+576, RStudio, Inc., Boston, MA) and R (http://www.R-project.org). A two-sided *p*-value of < 0.05 was deemed statistically significant.

‘

## 3. Results

### 3.1. Characteristics of patients with SSNHL based on their initial hearing level

**Figure 2:**
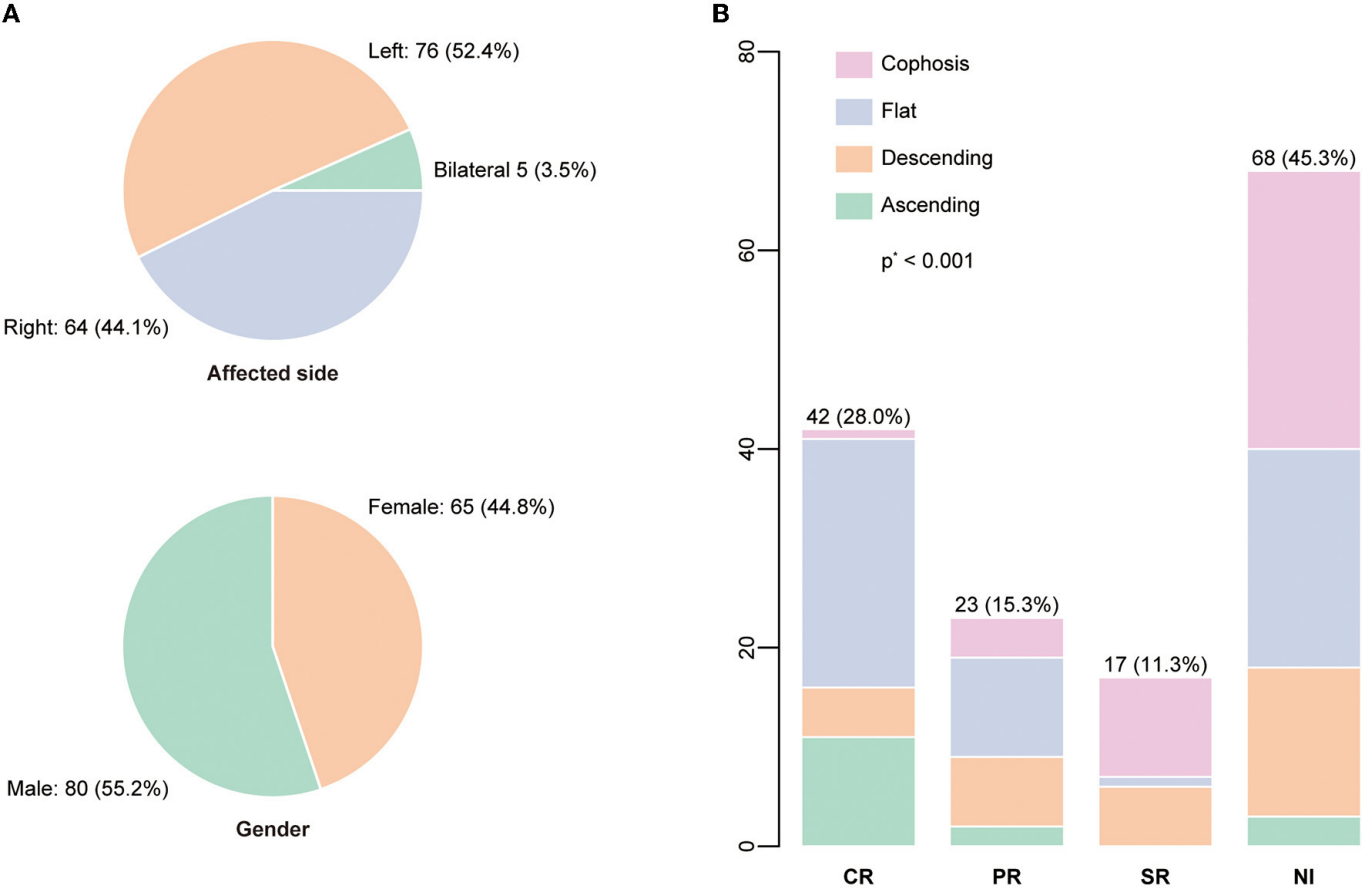
**(A)** The distributions of the affected side and gender were displayed. **(B)** The audiogram configuration distribution according to the recovery status based on Siegel's criteria. *P*-value was calculated by the Kruskal-Wallis test.

Table 1 summarizes the demographic and clinical characteristics and hearing outcomes for all enrolled patients, according to their baseline hearing level. The study comprised 145 patients (150 ears), of whom 65 were women (44.8%). The characteristics of the affected side and gender are displayed in Figure 2A. At the commencement of SSNHL, tinnitus was present in more than half of the patients (74/145, 51.0%). In contrast, only 30 patients had vertigo (20.7%), with a statistical difference between the baseline hearing groups (*P* = 0.021). Regarding the configurations of the initial audiogram, the flat configuration was the most common (58/150, 38.7%), and a significant difference in the initial hearing was detected among groups (*P* = 0.001). Eighty-one patients had complete blood count (CBC) test results, and 83 patients had coagulation factor test findings. The study found that patients with profound initial hearing loss had a significantly lower absolute lymphocyte count compared to those without [the profound group: 1.3 (1.0–2.0); the non-profound: 2.1 (1.4–2.6); *p* = 0.004]. The patients in the profound group had a higher level of PLR and a lower level of monocytes than their counterparts with less severe hearing loss.

**Table 1:** Clinical characteristics and recovery situation grouped by the initial hearing level.

		**Initial hearing level**		
	**Overall (*****N*** = **145)**	**Not profound (*****N*** = **90)**	**Profound (*****N*** = **55)**	***P***
Age (y)^*^	14.0 (11.0–16.0)	14.0 (11.0–17.0)	13.0 (11.0–16.0)	0.260^a^
Sex F:M^*^	65:80	41:49	24:31	0.864^b^
**Side of SSNHL**				
Unilateral: bilateral^*^	140:5	85:5	55:0	0.157^b^
L:R (150 ears)	81:69	52:43	29:26	0.866^b^
**Accompanying symptoms**				
Tinnitus^*^	74 (51.0%)	47 (52.2%)	27 (49.1%)	0.735^b^
Vertigo^*^	30 (20.7%)	13 (14.4%)	17 (30.9%)	0.021^b^
Onset of treatment^*^ (*N* = 124)	4.0 (3.0–10.0)	5.0 (3.0–10.0)	4.0 (2.0–8.5)	0.628^a^
Initial configuration (150 ears)				0.001^b^
Ascending	16 (10.7%)	16 (16.8%)	0 (0%)	
Descending	33 (22.0%)	29 (30.5%)	4 (7.3%)	
Flat	58 (38.7%)	50 (52.6%)	8 (14.5%)	
Cophosis	43 (28.7%)	0 (0%)	43 (78.2%)	
**Complete blood count (*****N*** = **81)**				
Neutrophil (10^9^/L)^*^	5.8 (4.8–7.2)	5.7 (4.6–7.5)	5.8 (5.1–6.8)	0.737^a^
Lymphocyte (10^9^/L)^*^	1.8 (1.2–2.5)	2.1 (1.4–2.6)	1.3 (1.0–2.0)	0.004^a^
Monocyte (10^9^/L)^*^	0.4 (0.2–0.6)	0.5 (0.3–0.7)	0.3 (0.1–0.5)	0.009^a^
Platelet (10^9^/L)^*^	297.6 (72.4)	303.1 (67.5)	288.8 (80.2)	0.414^c^
MPV (fl)^*^	10 (1.2)	9.8 (1.1)	10.3 (1.4)	0.122^c^
NLR^*^	3.5 (2.0–5.3)	3.1 (1.8–5.1)	4.7 (2.4–5.6)	0.066^a^
PLR^*^	167.6 (110.2–234.4)	142.2 (105.2–216.8)	218.7 (126.2–263.4)	0.041^a^
**Coagulation function (*****N*** = **83)**				
APTT (s)^*^	29.0 (12.2–36.6)	28.1 (12.2–36.3)	30.2 (12.3–36.7)	0.856^a^
FIB (g)^*^	2.3 (2.0–2.7)	2.4 (2.1–2.7)	2.2 (1.7–2.6)	0.148^a^
PT (s)^*^	14.1 (12.9–23.1)	13.7 (12.9–23.3)	14.4 (13.1–21.0)	0.592^a^
TT (s)^*^	17.6 (16.8–18.7)	17.6 (17.0–18.4)	17.7 (16.7–20.1)	0.637^a^
Follow-up days^*^	14.0 (12.0–90.0)	14.0 (11.2–90.0)	14.0 (13.5–76.5)	0.667^a^
**Hearing recovery (150 ears)**				
Recovery^#^	65 (43.3%)	56 (58.9%)	9 (16.4%)	< 0.001^b^
Hearing gain (dB HL)	21.5 (7.1–35.8)	21.7 (8.3–35.8)	19.2 (6.3–36.7)	0.641^a^
Final hearing (dB HL)	42.7 (19.4–75.8)	28.3 (15.8–46.2)	81.7 (55.0–95.4)	< 0.001^a^

Continuous variables were presented as mean (standard deviation) for normal distribution or medians (interquartile range) for non-normal distribution. Categorical variables were presented as *n* (%).

Subjects with one or more affected ears estimated as profound level hearing loss (average hearing threshold ≥ 80 dBHL) were assigned to the profound group.

Hearing gain and final hearing were evaluated by the hearing thresholds at frequencies affected (frequencies with initial hearing threshold > 20 dBHL).

^*^These were subject-specific covariates, while the rest were ear-specific covariates.

^#^According to Siegel's criteria, hearing recovery was defined as complete recovery (CR) and partial recovery (PR).

^a^Mann-Whitney *U*-test.

^b^Fischer's exact test.

^c^Student *t*-test.

MPV, mean platelet volume; NLR, neutrophil-to-lymphocyte ratio; PLR, platelet-to-lymphocyte ratio; APTT, activated partial thromboplastin time; FIB, fibrinogen; PT, prothrombin time test; TT, thrombin time.

### 3.2. Hearing outcome prognostic factor analysis using a subgroup comparison

**Figure 3:**
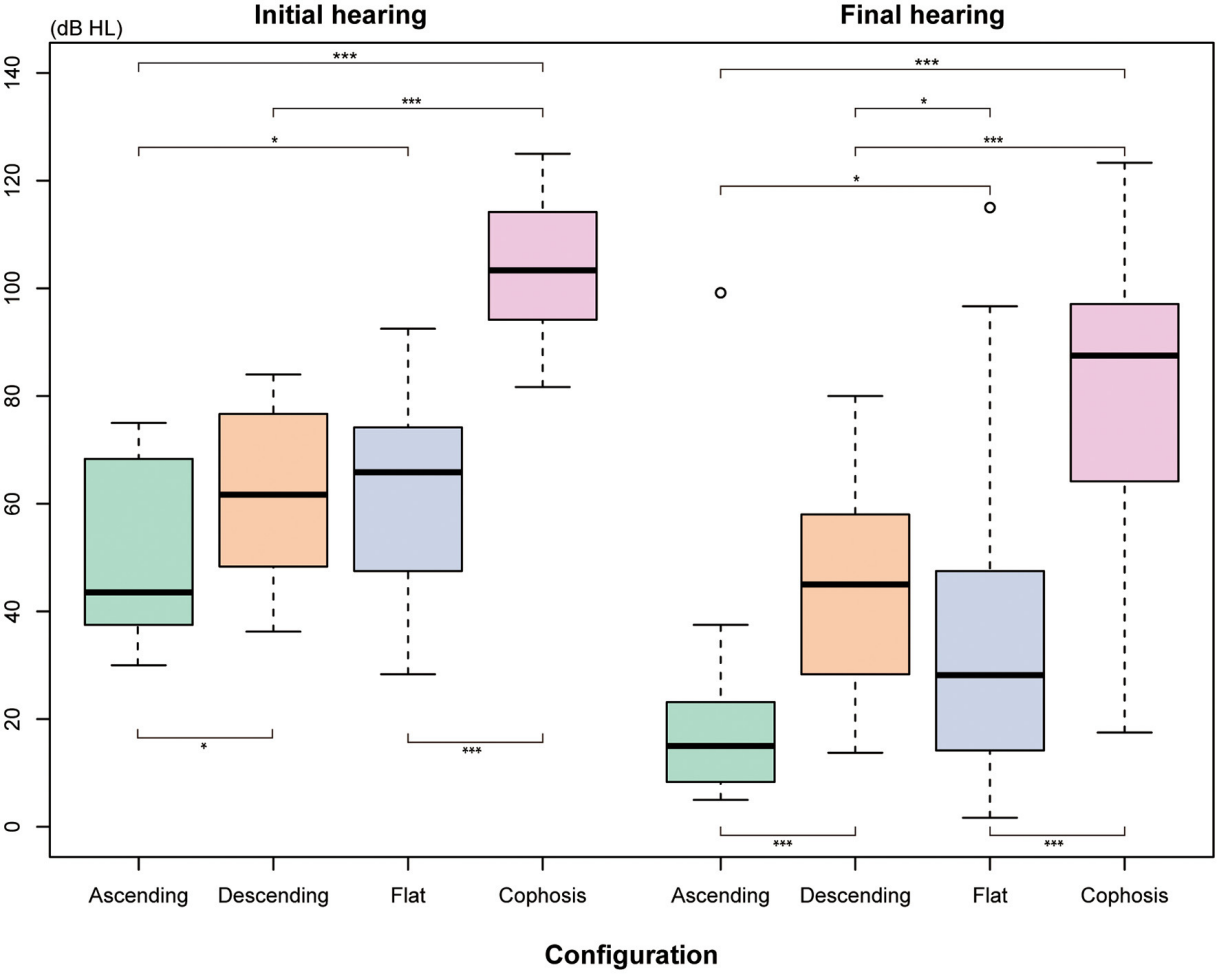
The means thresholds of the initial and final hearing with different audiogram configurations. The Mann-Whitney *U*-test was used for comparison. *Indicates that *P* ≤ 0.05. ***Indicates that *P* ≤ 0.001.

Figure 2B depicts the percentage and number of ears for each Siegel's recovery grade. Tinnitus was reported by 61.3% (38/62) of patients in the recovery group and 43.3% (36/83) in the patient group without recovery. The difference in accompanying tinnitus between the two outcome groups was statistically significant (*P* = 0.044) (Table 2). There were significant differences in the baseline threshold average between the non-recovery group and the recovery group [non-recovery: 84.0 (68.3–104.2); recovery: 60.8 (43.8–72.9), *P* = < 0.001] and in the subgroups separated by initial severity (*P* = 0.037). The configurations of the audiograms were significantly different across the groups (*P* = < 0.001). Patients with cophosis figures had significantly different recovery than those with descending (*P* = 0.020), ascending (*P* < 0.001), and flat figures (*P* < 0.001). Patients with descending figures had significantly different recovery than those with ascending (*P* = 0.011) and flat figures (*P* = 0.038) after *post-hoc* comparison. The thresholds for the initial and final hearing comparison, sorted by the initial configuration, are shown in Figure 3. There were significant differences in the final hearing between the cophosis configuration and the other three groups, respectively (ascending: *P* = < 0.001; descending: *P* = < 0.001; flat: *P* = < 0.001). A worse final hearing was found in patients with descending figures than with ascending (*P* = < 0.001) and flat (*P* = 0.016) figures, respectively. The threshold for a final hearing with ascending figures was similarly lower than for that with the flat figure (*P* = 0.019). No statistical difference was found in complete blood cell and coagulation test biomarkers across the recovery group and the non-recovery group.

**Table 2:** Clinical characteristics, laboratory tests, and audiograms related to hearing recovery.

	**Hearing outcomes**		
	**Non-recovery (*****N*** = **83)**	**Recovery**^#^ **(*****N*** = **62)**	***P***
Age (y)^*^	14.0 (11.0–16.0)	14.0 (11.0–16.8)	0.386^a^
Sex F:M^*^	40:43	25:37	0.400^b^
**Side of SSNHL**			
Unilateral: bilateral^*^	81:2	59:3	0.651^b^
L:R (150 ears)	46:39	35:30	1.000^b^
**Accompanying symptoms**			
Tinnitus^*^	36 (43.4%)	38 (61.3%)	0.044^b^
Vertigo^*^	19 (22.9%)	11 (17.7%)	0.536^b^
Onset of treatment^*^ (*N* = 124)	5.0 (3.0–12.0)	4.0 (2.0–7.0)	0.359^a^
Follow-up^*^	14.0 (12.0–54.0)	14.0 (13.2–90.0)	0.333^a^
**Baseline hearing profiles (150 ears)**			
Baseline threshold average (dB HL)	84.0 (68.3–104.2)	60.8 (43.8–72.9)	< 0.001^a^
Initial severity			0.037^d^
Mild	7 (8.2%)	15 (23.1%)	
Moderate	11 (12.9%)	20 (30.8%)	
Severe	21 (24.7%)	21 (32.3%)	
Profound	46 (54.1%)	9 (13.8%)	^e^
Initial configuration			< 0.001^b^
Ascending	3 (3.5%)	13 (20.0%)	
Descending	21 (24.7%)	12 (18.5%)	^f^
Flat	23 (27.1%)	35 (53.8%)	
Cophosis	38 (44.7%)	5 (7.7%)	^g^
**Complete blood count (*****N*** = **81)**			
Neutrophil (10^9^/L)^*^	5.6 (4.7–6.8)	6.0 (5.2–7.8)	0.197^a^
Lymphocyte (10^9^/L)^*^	1.5 (1.1–2.3)	2.1 (1.4–2.6)	0.057^a^
Monocyte (10^9^/L)^*^	0.4 (0.1–0.5)	0.5 (0.3–0.6)	0.224^a^
Platelet (10^9^/L)^*^	296.7 (79.7)	298.5 (65.9)	0.913^c^
MPV (fl)^*^	10.2 (1.4)	9.8 (1.1)	0.092^c^
NLR^*^	4.4 (2.1–5.6)	3.1 (1.8–5.2)	0.232^a^
PLR^*^	184.1 (132.3–247.9)	142.2 (101.8–223.5)	0.129^a^
**Coagulation function (*****N*** = **83)**			
APTT (s)^*^	32.0 (13.1–37.0)	26.3 (12.1–35.8)	0.156^a^
FIB (g)^*^	2.4 (1.9–2.8)	2.3 (2.0–2.6)	0.543^a^
PT (s)^*^	13.9 (12.9–16.6)	14.8 (13.1–23.3)	0.312^a^
TT (s)^*^	17.6 (16.7–19.5)	17.7 (16.9–18.4)	0.876^a^

Continuous variables were presented as mean (standard deviation) for normal distribution or medians (interquartile range) for non-normal distribution. Categorical variables were presented as *n* (%).

The baseline threshold average was evaluated by the hearing thresholds at frequencies affected (frequencies with initial hearing threshold > 20 dBHL).

Subjects with one or more affected ears estimated as not recovered based on Siegel's criteria were assigned to the non-recovery group.

^*^These were subject-specific covariates, while the rest were ear-specific covariates.

^#^According to Siegel's criteria, hearing recovery was defined as CR and PR.

^a^Mann-Whitney *U*-test.

^b^Fischer's exact test.

^c^Student *t*-test.

^d^Kruskal-Wallis test.

^e^Significant differences were found between profound and the other three groups after a *post-hoc* comparison in initial severity grouping.

^f^Significant differences were found between descending and the other three groups after a *post-hoc* comparison in initial configuration grouping.

^g^Significant differences were found between cophosis and the other three groups after a *post-hoc* comparison in initial configuration grouping.

MPV, mean platelet volume; NLR, neutrophil-to-lymphocyte ratio; PLR, platelet-to-lymphocyte ratio; APTT, activated partial thromboplastin time; FIB, fibrinogen; PT, prothrombin time test; TT, thrombin time.

### 3.3. Factors related to initial hearing and final hearing which were analyzed using the multivariate regression models

**Figure 4:**
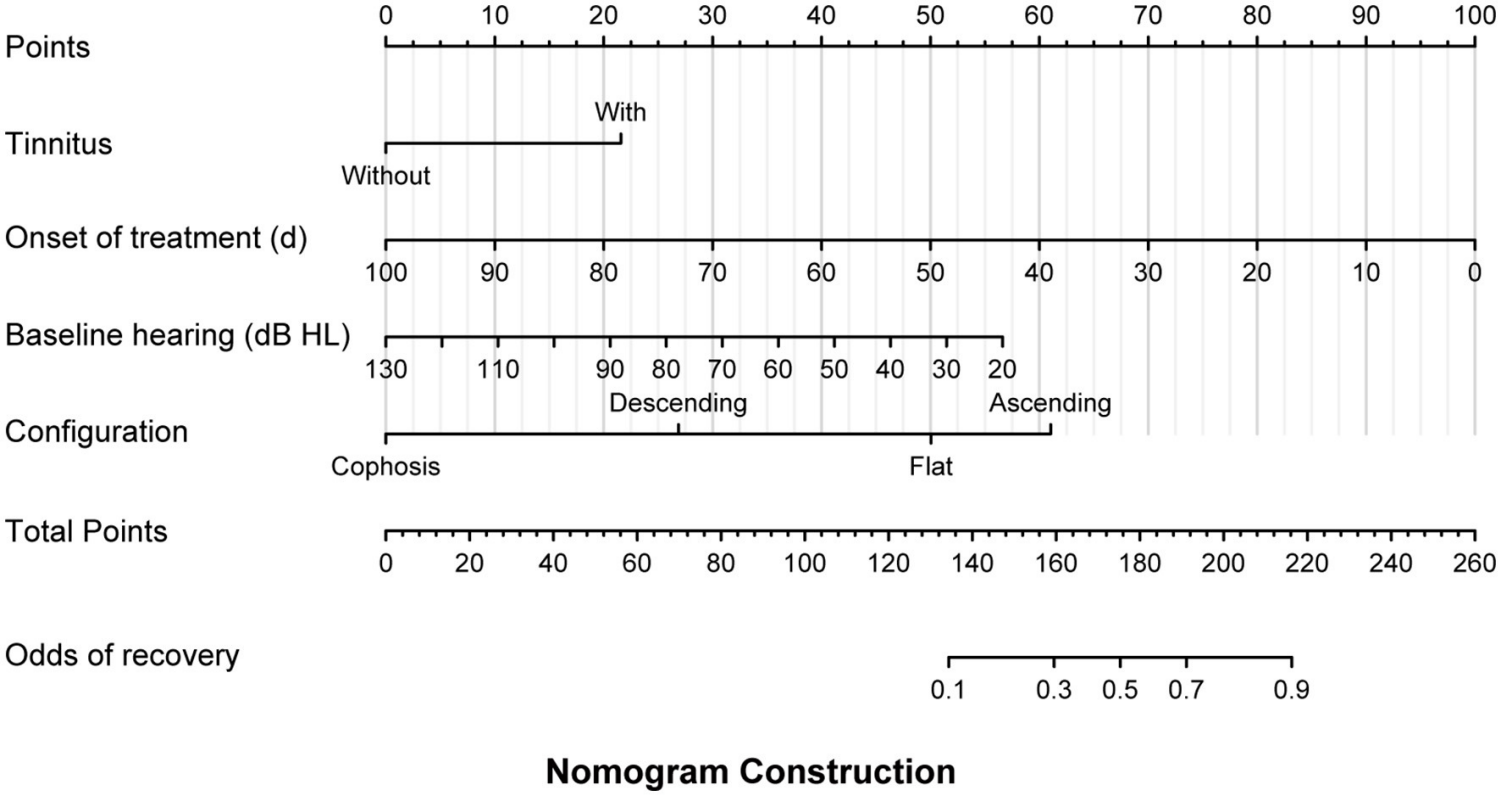
A multivariate logistic model was used to develop a prognostic nomogram for pediatric patients with sudden sensorineural hearing loss (SSNHL), which incorporated the odds of recovery as the response variable and independent variables including the presence of tinnitus, the onset of treatment, baseline hearing threshold, and audiogram configuration. To make a prediction regarding the probability of patient recovery in accordance with Siegel's criteria, the model identified the patient's values along each axis first. Subsequently, a vertical line was drawn upward from each value to the 'points' axis to determine the number of points generated by each variable. The points generated by all variables should then be summed to arrive at the total points line. Finally, a vertical line should be drawn down from this point, thereby providing the odds of recovery.

In the linear models, the factors related to the initial hearing level included vertigo (β = 13.932, 95% CI: 4.082–23.782, *P* = 0.007), lymphocyte count (β = −6.686, 95% CI: −10.919 to −2.454, *P* = 0.003) (Table 3), and independent predictors for the threshold for the final hearing, which comprised the onset of therapy (β = 0.313 95% CI: 0.125–0.502, *P* = 0.001), baseline hearing (β = 0.772, 95% CI: 0.563–0.980, *P* = < 0.001), and configurations (Table 4). In the multivariate logistic model adjusted for age, sex, and time to the onset of treatment (Table 5), the patients with ascending and flat audiograms were more likely to recover than the patients with descending audiograms (ascending: OR 8.168, 95% CI 1.450–70.143, *P* = 0.029; flat: OR 3.966, 95% CI 1.341–12.651, *P* = 0.015). The odds of recovery were 3.2 times higher for patients with tinnitus than for those without tinnitus (OR 3.222, 95% CI 1.241–8.907, *P* = 0.019). The threshold for baseline hearing (OR 0.968, 95% CI 0.936–0.998, *P* = 0.047) and the onset of therapy (OR 0.942, 95% CI 0.890–0.977, *P* = 0.010) were negatively associated with the odds of recovery. To predict the hearing outcomes of pediatric patients with SSNHL, a nomogram was established by incorporating the following parameters: tinnitus, the onset of therapy, baseline hearing, and configuration (Figure 4).

**Table 3:** Correlation between baseline characteristics and threshold of the initial hearing.

	***B***	**SE**	**β**	**Lower limit**	**Higher limit**	***P***
Age (y)	−1.134	0.699	−0.161	−2.503	0.235	0.109
Sex male	10.700	4.702	0.225	1.484	19.916	0.026
Vertigo	13.932	5.026	0.273	4.082	23.782	0.007
Lymphocyte (10^9^/L)	−6.686	2.159	−0.306	−10.919	−2.454	0.003

B, unstandardized coefficient; SE, standard error; β, standardized coefficient; lower limit, lower limit of 95% confidence interval for unstandardized coefficient; higher limit, higher limit of 95% confidence interval for unstandardized coefficient.

The correlation was analyzed by multivariate linear regression analysis adjusted by age and sex.

**Table 4:** Correlation between baseline characteristics and threshold of the final hearing.

	***B***	**SE**	**β**	**Lower limit**	**Higher limit**	***P***
Age (y)	−0.541	0.523	−0.056	−1.565	0.484	0.303
Sex men	−5.252	3.357	−0.079	−11.832	1.329	0.120
Tinnitus	−6.232	3.427	−0.094	−12.949	0.485	0.072
Onset of treatment (d)	0.313	0.096	0.165	0.125	0.502	0.001
Baseline hearing (dB HL)	0.772	0.106	0.601	0.563	0.980	< 0.001
**Configuration**						
Descending	Ref					
Ascending	−16.123	6.364	−0.153	−28.596	−3.650	0.013
Flat	−12.182	4.460	−0.180	−20.924	−3.440	0.007
Cophosis	7.037	6.561	0.096	−5.822	19.896	0.286

B, unstandardized coefficient; SE, standard error; β, standardized coefficient; lower limit, lower limit of 95% confidence interval for unstandardized coefficient; higher limit, higher limit of 95% confidence interval for unstandardized coefficient.

The correlation was analyzed by multivariate regression analysis adjusted by age, sex, and the onset of treatment.

**Table 5:** Correlation between baseline characteristics and hearing recovery (recovery vs. non-recovery).

	***B***	**Odds ratio**	**Lower limit**	**Higher limit**	***P***
Age (y)	0.006	1.006	0.866	1.168	0.938
Sex men	0.889	2.433	0.923	6.899	0.080
Tinnitus	1.170	3.222	1.241	8.907	0.019
Onset of treatment (*d*)	−0.059	0.942	0.890	0.977	0.010
Baseline hearing (dB HL)	−0.032	0.968	0.936	0.998	0.047
**Configuration**					
Descending	Ref				
Ascending	2.100	8.168	1.450	70.143	0.029
Flat	1.378	3.966	1.341	12.651	0.015
Cophosis	−1.197	0.302	0.032	2.153	0.250

B, unstandardized coefficient; lower limit, lower limit of 95% confidence interval for odds ratio; higher limit, higher limit of 95% confidence interval for the odds ratio.

The correlation was analyzed by multivariate logistic regression analysis adjusted by age, sex, and the onset of treatment.

According to Siegel's criteria, hearing recovery was defined as complete recovery (CR) and partial recovery (PR).

## 4. Discussion

The present bi-center study found some predictive factors of hearing outcomes in pediatric patients with SSNHL, including tinnitus, the time elapsed from the onset of the symptoms to the commencement of the treatment, initial audiogram configuration, and hearing levels. We observed a higher proportion of accompanying vertigo, a greater degree of inflammatory biomarkers (lower the concentration of lymphocytes. and a higher level of PLR), and worse hearing outcomes (lower recovery rate and increased final hearing thresholds) in patients with more severe initial hearing loss. Meanwhile, according to the multivariate linear regression model, we developed a nomograph as a simple predictive tool for the final hearing threshold.

The coexisting symptoms of vertigo (29–56%) and tinnitus (41–90%) were frequently observed in adult patients with SSNHL (19, 20). The incidence rate of both symptoms is similar in pediatric patients with SSNHL, as reported in a previous study (21). We noticed that tinnitus is independently associated with better hearing outcomes in pediatric patients with SSNHL; this finding is in line with earlier research in both children and adults (6, 22–25). Based on the multivariate analysis, pediatric patients with SSNHL and tinnitus generally have a 3.2-fold improvement in their recovery odds compared to those without. A recent study revealed an underlying mechanism for different cortical activity patterns in adult SSNHL patients with and without tinnitus (26), which may also be applicable to the child population. With regards to the presence of vertigo, some differences were observed. It was consistently reported to be detrimental to hearing recovery in adult patients (27). In contrast, it is still controversial in the studies on children and adolescent patients (6, 24, 25, 28–30). Our findings suggest that the baseline hearing severity but not the hearing outcome was independently related to the presence of vertigo. This finding is consistent with a previous study by Liu et al. (31), finding that patients with SSNHL with vertigo might suffer from a more severe cochlear and vestibular impairment, as indicated by vestibular function testing. It indicates that vertigo may indicate a more serious condition in pediatric patients with SSNHL, which could be a potential prognostic factor.

In terms of audiograms, we observed that patients with ascending and flat configurations had lower thresholds for the final hearing and greater recovery odds than patients with descending figures; these findings were consistent with the conclusions drawn by Qian et al. (30) and Chen et al. (6). However, Kim et al. (25) pointed out that although the decreasing figure was a good predictor, they had only found it in six individuals. According to the literature on adults, low-frequency hearing loss was confirmed to be the positive predictor, whereas the descending figure was the opposite indicator (19, 22, 32, 33). In addition, mid-frequency hearing loss, categorized under the flat figure in our analysis, was also referred to as a positive prognostic factor (34). Moreover, it appeared that for children, the association between configurations and hearing recovery was comparable with that for adults. Regarding the descending configuration, it was found that the hair cells at the base of the cochlea were delicate and that regaining high-frequency hearing function was more difficult (35–38). In addition, it has been reported that high-frequency hearing loss can go unnoticed in children, leading to delays in initiating therapy (39). This could explain why the descending configuration in pediatric patients with SSNHA is associated with poor recovery.

This study's results found that the lymphocyte count correlated with the initial hearing thresholds, with a marginally higher value in patients who recovered. Although a low lymphocyte count was identified as a risk factor and a poor prognostic factor in adults (13, 14), recent study reported inconsistent findings for children because of the small sample size (10–12). As an indicator of inflammation (40), low lymphocyte count in SSNHL was assumed to be caused by increased T lymphocyte extravasation from the blood vessel (13). Meanwhile, virus-induced immunosuppression might be another inflammation-related cause of decreased lymphocyte count (7, 8). According to our findings, PLR, another inflammatory biomarker (41), was significantly higher in the patients with profound initial hearing, and NLR had the same tendency but with a marginal significance. Several inflammatory agents were also seen to cause cochlear damage (42, 43). These findings suggested that the extent of systemic inflammation might be associated with the severity of SSNHL in children.

In the current study, we observed that among the ears studied, 36.7% had profound initial hearing, and among these ears, 45.3% showed no improvement. The findings were similar to the conclusion drawn by a recent meta-analysis (21), with 36.7% of ears having profound hearing loss and 46.7% showing no improvement. However, substantial heterogeneity was present because of the relatively small sample, and the criteria in each research varied. Although previous studies indicated a higher complete recovery rate in children compared to adults, one of them defined adults as older than 15 years, and the other comprised a small sample of fewer than 40 children (6, 7). Conversely, age < 15 years was regarded as a sign of poor prognosis in other literature (44, 45). Our study found no statistical significance in the correlation of age with severity and outcomes. The relationship between age and outcomes in the pediatric population would benefit from more precise examinations and analyses.

To the best of our knowledge, our study on SSNHL in children is one of the largest of its kind, with a sample size of 145 patients. Furthermore, we conducted a bi-center investigation, which increases the representativeness of the target population and reduces individual bias compared to single-center studies. Nonetheless, there are several drawbacks. First, because of the respective research design, it was impossible to confirm causal relationships between the investigated factors and the severity or outcomes. Second, the investigation was exploratory without a scientistic hypothesis or pivot statistical estimate—no sample size calculation to ensure a significant statistical power. Meanwhile, due to the low prevalence of SSNHL in children, only a portion of the patients had complete blood counts and coagulation tests available. Third, only the total lymphocyte count was determined; no subtype analysis was performed. Therefore, further research is warranted to focus on the potential biomarkers in children to demonstrate whether these biomarkers differ from those in adults.

In conclusion, it was an exploratory study on pediatric patients with SSNHL. Some factors, including accompanying tinnitus, the severity of the initial hearing loss, the onset of therapy, and the configuration of audiograms, might be related to the prognosis of pediatric patients with SSNHL. Meanwhile, the incidence of vertigo, lower lymphocytes, and higher PLR were associated with poor hearing severity. Further research is needed to establish a more accurate understanding of the relationship between pediatric SSNHL and its underlying mechanisms, given the diversity of clinical trials involving adults and children.

## Data availability statement

The raw data supporting the conclusions of this article will be made available by the authors, without undue reservation.

## Ethics statement

The studies involving human participants were reviewed and approved by Ethics Committee, Tongji Hospital, Tongji Medical College, Huazhong University of Science and Technology, Wuhan, PR China and Ethics Committee, The First People's Hospital of Foshan, Foshan, PR China. Written informed consent from the participants' legal guardian/next of kin was not required to participate in this study in accordance with the national legislation and the institutional requirements.

## Author contributions

DB contributed to the study design, data analysis, and manuscript writing. YL and XZ contributed to the study design, data collection and analysis, and manuscript writing. ZD and DD contributed to the data analysis, collection, and holding. All authors contributed to the article and approved the submitted version.
